# A Mitochondrial Membrane Exopolyphosphatase Is Modulated by, and Plays a Role in, the Energy Metabolism of Hard Tick *Rhipicephalus (Boophilus*) *microplus* Embryos

**DOI:** 10.3390/ijms12063525

**Published:** 2011-06-03

**Authors:** Eldo Campos, Arnoldo R. Façanha, Evenilton P. Costa, Amanda Fraga, Jorge Moraes, Itabajara da Silva Vaz, Aoi Masuda, Carlos Logullo

**Affiliations:** 1 Laboratório Integrado de Bioquímica—Hatisaburo Masuda, UFRJ, Polo Barreto, Rua Rotary Club s/n, São José do Barreto, Macaé, RJ, C.P. 119.331, CEP 27971–220, Brazil; E-Mails: eldocampos@macae.ufrj.br (E.C.); manda_fraga@yahoo.com.br (A.F.); jorgemoraes@bioqmed.ufrj.br (J.M.); 2 Instituto Nacional de Ciência e Tecnologia—Entomologia Molecular, Rio de Janeiro, RJ, CEP 21941–590, Brazil; E-Mails: arnoldo@uenf.br (A.R.F.); itabajara.vaz@ufrgs.br (I.d.S.V.); aoi@cbiot.ufrgs.br (A.M.); 3 Laboratório de Química e Função de Proteínas e Peptídeos, Laboratório de Biologia Celular e Tecidual and Unidade de Experimentação Animal–CBB–UENF, Avenida Alberto Lamego, 2000, Horto, Campos dos Goytacazes, RJ, CEP 28015–620, Brazil; E-Mail: eveniltonpessoa@yahoo.com.br; 4 Centro de Biotecnologia e Faculdade de Veterinária, UFRGS, Avenida Bento Gonçalves, 9090, Porto Alegre, RS, C.P. 15005, CEP 91501–970, Brazil; 5 Centro de Biotecnologia, UFRGS, Avenida Bento Gonçalves, 9500, prédio 43421, Porto Alegre, RS, C.P. 15005, CEP 91501–970, Brazil

**Keywords:** inorganic polyphosphate, respiration, membrane exopolyphosphatase, arthropod, energy metabolism

## Abstract

The physiological roles of polyphosphates (polyP) recently found in arthropod mitochondria remain obscure. Here, the relationship between the mitochondrial membrane exopolyphosphatase (PPX) and the energy metabolism of hard tick *Rhipicephalus microplus* embryos are investigated. Mitochondrial respiration was activated by adenosine diphosphate using polyP as the only source of inorganic phosphate (P_i_) and this activation was much greater using polyP_3_ than polyP_15_. After mitochondrial subfractionation, most of the PPX activity was recovered in the membrane fraction and its kinetic analysis revealed that the affinity for polyP_3_ was 10 times stronger than that for polyP_15_. Membrane PPX activity was also increased in the presence of the respiratory substrate pyruvic acid and after addition of the protonophore carbonyl cyanide-p-trifluoromethoxyphenylhydrazone. Furthermore, these stimulatory effects disappeared upon addition of the cytochrome oxidase inhibitor potassium cyanide and the activity was completely inhibited by 20 μg/mL heparin. The activity was either increased or decreased by 50% upon addition of dithiothreitol or hydrogen peroxide, respectively, suggesting redox regulation. These results indicate a PPX activity that is regulated during mitochondrial respiration and that plays a role in adenosine-5′-triphosphate synthesis in hard tick embryos.

## 1. Introduction

Inorganic polyphosphates (polyP) are long chains of a few to several hundred phosphate residues linked by phosphoanhydride bonds. PolyP are ubiquitously found in all cell types examined to date and have been demonstrated to play diverse roles depending on the cell type and circumstances [1,2].

The biological roles played by polyP have been studied most extensively in prokaryotes and unicellular eukaryotes, where they have been shown to regulate many biochemical processes, including metabolism and transport of inorganic phosphate (P_i_), cation sequestration and storage [1], formation of membrane channels [3,4], involvement in cell envelope formation and function in bacterial pathogenesis [5,6], regulation of gene and enzyme activities [7], activation of Lon proteases [8], and KcsA channel regulation [9]. Conversely, polyP functions have not been extensively investigated in higher eukaryotes, although some functions have been described, such as activation of TOR kinase [10], involvement in blood coagulation [11], and apoptosis [12–14]. Recently, we have reported the first evidence that polyP also play key roles in arthropods and have described a mitochondrial and a nuclear exopolyphosphatase involved in metabolism during embryogenesis of the hard tick *Rhipicephalus microplus* [15,16].

Exopolyphosphatase (PPX) splits P_i_ off the end of a polyP chain and represents one of the main enzyme types responsible for polyP hydrolysis [1]. PolyP metabolism in eukaryotic cells shows specific peculiarities for different cellular compartments, including mitochondria [17]. At least two PPXs have been identified in *Saccharomyces cerevisiae* mitochondria [18], which possess their own polyP pool [19] that was described as a potential P_i_ source for oxidative phosphorylation [20], these PPX are well characterized (PPX; polyphosphate phosphohydrolase; EC 3.6.1.11), however in higher eukaryotes the protein responsible for PPX activity is not known.

The present study is focused on *R. microplus*, a one-host tick that causes major losses to bovine herds, particularly in tropical regions; thus, major efforts have been directed toward developing immunoprophylactic tick-control tools [21]. Ticks are vectors of parasites that cause hemoparasitic diseases and are endemic in many cattle production areas [22]. *R. microplus* has one only host throughout its three life stages, usually a bovine host, and a long feeding period (approximately 21 days). Female ticks, after engorgement, drop off the host and initiate oviposition approximately three days later. Being an oviparous animal, embryogenesis occurs in the absence of exogenous nutrients and maternal nutrients are packaged in oocytes and stored mostly as yolk granules. Hatching occurs approximately 21 days after egg-laying and the emerging larvae can survive several weeks before finding a host, using the remaining yolk as the only energy source [23].

Here, we investigated mitochondrial membrane PPX regulation during mitochondrial respiration in *R. microplus* embryos, revealing an important role for this enzyme in tick energy metabolism.

## 2. Results and Discussion

Although the first evidence for the presence of polyP in mammalian cells were obtained long ago [24], since then relatively few studies have addressed their physiological roles in animal cells [1,10,11,13–16,25,26].

Early *R. microplus* embryonic stages are similar to those of *D. melanogaster* and mosquitoes [27,28]. Tick embryogenesis is characterized by formation of a non-cellular syncitium up to day 4. Thereafter, the embryo becomes a multicellular organism and initiates organogenesis [29]. Previously, we have found that during egg segmentation (9th day after oviposition), a strong mitochondrial PPX activity exists [15]. Here, we provide evidence that mitochondrial membrane PPX plays a role in energy metabolism of *R. microplus* during embryo development.

### 2.1. Characterization of Isolated Mitochondria

Mitochondria from tick embryos in the segmentation stage (9th day after oviposition) were isolated and cellular respiration was measured using pyruvate as the substrate. Oxygen consumption was 30 nmol/min·mg protein and the RCR (respiratory control ratio) was 6.5. The process was KCN- and oligomycin-sensitive (Table 1).

Respiratory parameters of mitochondria in the presence of pyruvate (5 mM). The rates of respiration in State 3 (phosphorylating respiratory rate) and in State 4 (non-phosphorylating respiratory rate) are expressed as nmol O_2_/min·mg protein. The results represent mean ± SD. of three independent experiments.

### 2.2. Influence of Membrane PPX in Mitochondrial Respiration

**Figure 1:**
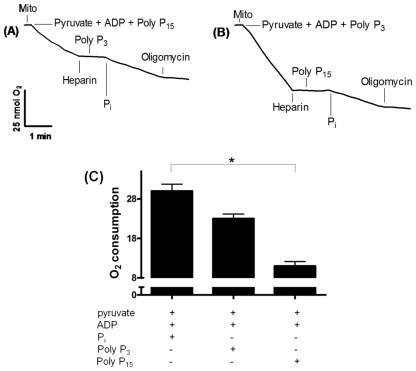
Involvement of membrane PPX in mitochondrial respiration. Oxygen consumption was monitored using a reaction buffer in the absence of a P_i_ source in eggs on the 9th day of development. In **(A)** and **(B)**, the addition of 1 mM ADP, 5 mM pyruvate, 0.5μM polyP_3 and 15_, 20 μg/mL heparin, 5 mM P_i_ and 0.5 μM oligomycin is represented in the figure. This experiment was repeated at least three times with different preparations, and this figure shows a representative experiment. In **(C)**, the oxygen consumption was quantified using 1 mM ADP, 5 mM P_i_, 5 mM pyruvate and 0.5μM polyP_3 and 15_. Asterisk (*) denotes the difference between population and the significance was determined by two way ANOVA test (Kruskal–Wallis).

We have previously demonstrated that polyP can be used as a P_i_ donor for adenosine-5′-triphosphate (ATP) synthesis in ticks [15]. To investigate the location of mitochondrial PPX, we assayed oxygen consumption using polyP_3_ and polyP_15_ as substrates and heparin as a PPX inhibitor. adenosine diphosphate-dependent mitochondrial oxygen consumption could be measured in the presence of polyP_3_ and polyP_15_ and in the absence of any other source of P_i_, supporting the hypothesis previously postulated that polyP can be used as a P_i_ donor for ATP synthesis [15]. However, this consumption was inhibited by heparin. Subsequently, oxygen consumption was recovered when 5 mM P_i_ was added, which was interrupted by addition of oligomycin, an ATP-synthase inhibitor (Figure 1A and 1B). Oxygen consumption was distinct using both polyP: no statistical difference was found using polyP_3_ compared with P_i_; otherwise, oxygen consumption was lower when polyP_15_ was used (Figure 1C).

**Figure 2:**
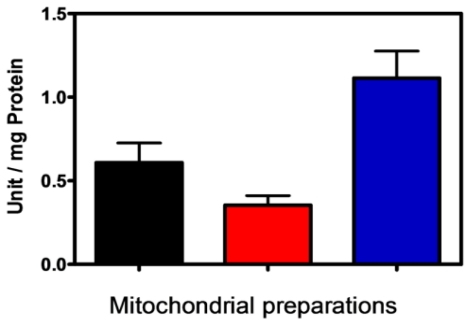
PPX activity in mitochondrial preparations. PPX activity was measured in mitochondria (black bar), soluble (red bar) and membrane fractions (blue bar) of the eggs on the 9th day of development using polyP_3_ as substrate. The activity was expressed as units per milligram of total protein and the results represent mean ± SD. of three independent experiments, in triplicate.

This new data suggest the existence of membrane PPX in this process due to the inhibition by heparin, which cannot cross the mitochondrial membrane, which has its active site oriented to the external face of the membrane. In fact, after subfractionation, the main PPX activity was recovered in the membrane fraction, supporting this hypothesis (Figure 2).

### 2.3. Characterization of PPX Activity in Mitochondrial Membrane Preparation

Exopolyphosphatases have been found in prokaryotes and eukaryotes, and although in bacteria these enzymes hydrolyze mostly high-molecular-weight polyP [17], at least some of the enzymes from *S. cerevisiae* and *Leishmania major* are more active in hydrolyzing short-chain polyP such as polyP_3_ [17,30].

To obtain insight into membrane PPX kinetics, the apparent *K_m_* was measured using polyP_3_ and polyP_15_ as substrates and results were expressed as the average of three independent experiments. Parameters obtained were very similar to those observed in crude mitochondria recently for our group [16]; the membrane PPX affinity for polyP_3_ was 10 times stronger than for polyP_15_ (Table 2). These results are in contrast to those found in a mitochondria membrane-bound PPX of *S. cerevisiae*, in which case the affinity was stronger for long-chain polyP [31]. However, the new data demonstrated that membrane PPX kinetics are in agreement with oxygen consumption that was much higher using polyP_3_ than polyP_15_. Heparin, an effective inhibitor of PPX [1], blocked the activity (Table 2). These results reinforce the coupling existing between this enzyme activity and mitochondrial ADP phosphorylation.

### 2.4. PPX Activity during Mitochondrial Respiration

**Figure 3:**
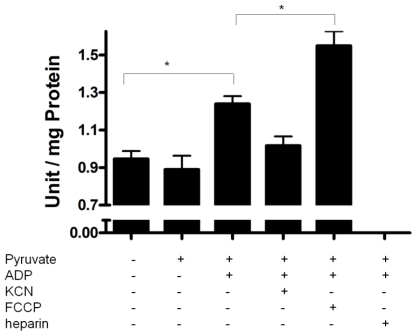
Regulation of mitochondrial PPX activity during mitochondrial respiration. PPX activity was measured in mitochondria of the eggs on the 9th day of development during mitochondrial respiration, using pyruvate as oxidative substrates, polyP_3_ as PPX substrate, KCN as inhibitor of the respiratory chain, FCCP as uncoupler and Heparin as PPX inhibitor. The activity was expressed as units per milligram of total protein and the results represent mean ± SD. of three independent experiments, in triplicate. Asterisk (*) denotes the difference between population and the significance was determined by two way ANOVA test (Kruskal–Wallis).

To further investigate regulation of membrane PPX during mitochondrial respiration, the activity was measured using pyruvate as the substrate and polyP as the only source of P_i_. During this assay, addition of a small amounts of ADP (0.2 mM) induces a state 3 followed by a state 4, when all the ADP was converted to ATP. Thus, during state 3, a balance exists between P_i_ release by PPX and ATP synthesis because PPX is measured by the amount of P_i_. Membrane PPX activity increased during mitochondrial respiration when pyruvate and ADP were added. This increase did not occur without ADP addition, indicating that PPX is stimulated during state 3 and the velocity of P_i_ release is higher than ATP synthesis. Indeed, the stimulatory effect was antagonized by KCN (decreased electron flux) addition and increased by FCCP (increased electron flux) (Figure 3), suggesting that membrane PPX could be modulated by electron flux. These data are in agreement with our previous work in which we demonstrated that mitochondrial PPX activity is regulated by energy demand [15]. Additionally, these findings are consistent with those of [32], who demonstrated that production and consumption of mitochondrial polyP depend on the activity of the oxidative phosphorylation machinery in mammalian cells. Furthermore, heparin inhibited PPX activity completely, reinforcing the role of membrane PPX during mitochondrial respiration and the respiration activation by membrane PPX activity indicates that PPX could be close to the site of ATP production.

Additionally, mitochondrial polyP can form polyP/Ca^2+^/PHB complexes [3] with ion-conducting properties similar to those of the native mitochondrial permeability transition pore [25]. Polyphosphatases localized in the membrane may not only degrade, but also synthesize polyP inside these complexes [31]. Recently, we demonstrated that synthesis of polyP occurs during embryogenesis of *R. microplus* in mitochondria, but not in nuclei [15,16]. As polyphosphate kinases have been found only in prokaryotes, the observation that polyP synthesis in ticks only occurs in the mitochondrial fraction supports the possibility that such synthesis probably occurs by the action of these complexes, as already suggested for other organisms [3,31,33,34].

### 2.5. Mitochondrial Membrane PPX Redox Sensitivity

**Figure 4:**
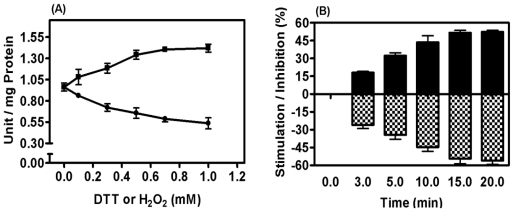
Redox regulation of membrane mitochondrial PPX. PPX activity was measured in mitochondria of the eggs on the 9th day of development using polyP_3_ as substrate. In **(A)**, the activity was measured in the presence of 0.1–1.0 mM of DTT (■) and H_2_O_2_ (●). In **(B)**, the mitochondria were treated with 1 mM DTT (black bar) and 1 mM H_2_O_2_ (hachured bar) for 0–20 min. The results represent mean ± SD. of three independent experiments, in triplicate.

Despite regulation of membrane PPX by increased or decreased electron flux, the sensitivity of this enzyme according to redox state using polyP_3_ as the substrate was evaluated. The influence of dithiothreitol (DTT) and hydrogen peroxide (H_2_O_2_) was investigated at different times and in a concentration range of 0.1 to 1.0 mM and PPX activity was stimulated and inhibited by 50%, respectively, suggesting that PPX is tightly regulated by redox state (Figure 4A and 4B).

## 3. Experimental Section

### 3.1. Ticks

Ticks were obtained from a colony maintained at the Faculdade de Veterinária, Universidade Federal do Rio Grande do Sul (UFRGS), Brazil. *R. microplus* (Acarina, Ixodidae) ticks from the Porto Alegre strain, free of parasites, were reared on calves obtained from a tick-free area. Engorged adult females were maintained in Petri dishes at 28 °C and 80% relative humidity upon completion of oviposition, which starts approximately three days after adult ticks drop off calves. Animals were treated in compliance with the UFRGS review committee for animal care.

### 3.2. Chemicals Materials

ADP, pyruvate, sodium phosphate glass type 15 (polyP_15_), sodium tripolyphosphate (polyP_3_), heparin, FCCP, oligomycin, KCN, DTT, H_2_O_2_ were purchased from Sigma Adrich. All other reagents were analytical grade.

### 3.3. Isolation of Mitochondria

The cell fractionation procedure used required large amounts of fresh eggs (at least 2 g) to obtain functionally active mitochondrial fractions. For characterization of mitochondrial fractions, eggs in the segmentation stage (9th day after oviposition) were used and mitochondria were isolated as previously described [15]. Isolation of the mitochondria membrane fraction was performed by sonication of freshly prepared mitochondria three times for 20 s at the maximal output using an MSE ultrasonic disintegrator. The suspension was centrifuged for 10 min at 12,000 × *g* to remove unbroken mitochondria. The supernatant was centrifuged at 100,000 × *g* for 60 min to yield the mitochondria membrane fraction. The supernatant was a soluble preparation of mitochondria, which included both the intermembrane space and matrix, and the pellet was a mix of inner and outer membranes.

### 3.4. PPX Assay and Kinetic Parameters

The reaction mixture consisted of 50 mM Tris-HCl buffer (pH 7.5) and 5 mM MgCl_2_, using 5 mM polyP_3_ or polyP_15_, as the substrate. Reactions were performed at 30 °C for various time periods. The P_i_ formed during the reaction was spectrophotometrically determined as previously described [35] by adding a solution of 0.5% ammonium molybdate, 0.35 M sulfuric acid, 0.5% sodium dodecyl sulfate, and 10% ascorbic acid. Measurements of absorbance at 750 nm were performed after 15 min. The enzyme amount liberating 1 μmoL of P_i_ per 1 min was defined as one unit of enzyme activity (U). Protein concentration was measured as described previously [36] using bovine serum albumin as a standard.

PPX activity during mitochondrial respiration was measured using a reaction mixture consisting of 50 mM Tris-HCl buffer (pH 7.2), 120 mM KCl, 1 mM EGTA, 5 mM MgCl_2_, and 0.2 mM adenosine diphosphate (ADP) in the absence of any P_i_ source. PolyP_3_ (0.5 μM) was used as a substrate for PPX activity and 5 mM pyruvate was used as an oxidative substrate. Potassium cyanide (KCN, 1 mM) and 20 μg/mL heparin were used to inhibit cytochrome oxidase and PPX activities, respectively, and 200 nM carbonyl cyanide-p-trifluoromethoxyphenylhydrazone (FCCP) was used as an uncoupler. The reaction was performed at 28 °C for 15 min [15].

Kinetic parameters were estimated by nonlinear regression analysis applied to the Michaelis-Menten equation using the program package supplied by GraphPad Prism 5.0.

### 3.5. Respiration Measurements

The rate of O_2_ uptake by mitochondria was estimated with a Clark oxygen electrode (YSI, mod. 5775, Yellow Springs, OH, USA). The calibration process was conducted using the initial O_2_ concentration of the medium as 100% O_2_-saturated buffer measured at 28 °C. Measurements were performed in 1.5 mL of reaction buffer containing 120 mM KCl, 1 mM EGTA, 0.2% bovine albumin, and 3 mM HEPES (pH 7.2) in the absence of any P_i_ source, containing 0.5 mg/mL of mitochondrial protein. After a 1-min equilibration period, mitochondrial respiration was started by addition of pyruvate to a final concentration of 5 mM. Each experiment was repeated at least three times with different mitochondrial preparations. Figure 1 shows a representative experiment and other additions are indicated in the figure legend [15]. Respiratory control ratio (RCR) values were obtained with isolated mitochondria by using pyruvate as the complex I substrate.

## 4. Conclusion

The ubiquity of polyP and the variation in its chain length, location, and metabolism indicate the relevant functions of this polymer, including those in animal systems. The present study clearly demonstrates that electron flux and redox state may exert some influence and be influenced by the activity of membrane PPX, suggesting that it plays a role in energy supply during *R. microplus* embryogenesis.

## Figures and Tables

**Table 1:** Mitochondria isolation from R*. microplus* embryos on the 9th day of embryogenesis.

	State 3	State 3	RCR
Mitochondria	30.2 ± 3.2	4.6 ± 0.7	6.5 ± 0.4

**Table 2:** Characterization of PPX activity in membrane preparation of mitochondria of *R. microplus* embryos on the 9th day of embryogenesis.

Substrates	Km (μM)	Vmax (μmol·min^−1^·mg protein^−1^)	Heparin (% inhibition)	O_2_ consumption (nmol.min^−1^·mg protein^−1^)
PolyP_3_	0.2	2.4	98	23.85 ± 2.06
PolyP_15_	2.2	1.1	98	11.44 ± 1.79
